# Hyper-Transcranial Alternating Current Stimulation: Experimental Manipulation of Inter-Brain Synchrony

**DOI:** 10.3389/fnhum.2017.00539

**Published:** 2017-11-08

**Authors:** Caroline Szymanski, Viktor Müller, Timothy R. Brick, Timo von Oertzen, Ulman Lindenberger

**Affiliations:** ^1^Center for Lifespan Psychology, Max Planck Institute for Human Development, Berlin, Germany; ^2^Berlin School of Mind and Brain, Humboldt University of Berlin, Berlin, Germany; ^3^Department of Human Development and Family Studies, Pennsylvania State University, State College, PA, United States; ^4^Department of Humanities, Universität der Bundeswehr München, München, Germany; ^5^European University Institute, Fiesole, Italy

**Keywords:** joint action, interpersonal coordination, hyperscanning, tACS

## Abstract

We walk together, we watch together, we win together: Interpersonally coordinated actions are omnipresent in everyday life, yet the associated neural mechanisms are not well understood. Available evidence suggests that the synchronization of oscillatory activity across brains may provide a mechanism for the temporal alignment of actions between two or more individuals. In an attempt to provide a direct test of this hypothesis, we applied transcranial alternating current stimulation simultaneously to two individuals (hyper-tACS) who were asked to drum in synchrony at a set pace. Thirty-eight female-female dyads performed the dyadic drumming in the course of 3 weeks under three different hyper-tACS stimulation conditions: same-phase-same-frequency; different-phase-different-frequency; sham. Based on available evidence and theoretical considerations, stimulation was applied over right frontal and parietal sites in the theta frequency range. We predicted that same-phase-same-frequency stimulation would improve interpersonal action coordination, expressed as the degree of synchrony in dyadic drumming, relative to the other two conditions. Contrary to expectations, both the same-phase-same-frequency and the different-phase-different-frequency conditions were associated with greater dyadic drumming asynchrony relative to the sham condition. No influence of hyper-tACS on behavioral performance was seen when participants were asked to drum separately in synchrony to a metronome. Individual and dyad preferred drumming tempo was also unaffected by hyper-tACS. We discuss limitations of the present version of the hyper-tACS paradigm, and suggest avenues for future research.

## Introduction

### Inter-Brain Synchronization during Joint Action

Joint actions abound in everyday life. When passing plates at the dinner table, when enjoying card games or when playing music together (Keller et al., 2014), we always need to coordinate our actions with others in time and space (Sebanz et al., 2006). Shared task representations have been suggested as the foundation of coordinated joint action (Knoblich et al., 2011). During joint action, humans appear to represent not only their own motor actions, but also the actions currently being performed and to be performed by their co-actors (Vesper et al., 2010). For example, cues prompting a co-actor to move activate neural processes associated with mental state attribution and motor inhibition (Ramnani and Miall, 2004; Tsai et al., 2006). Conceptually speaking, multiple persons engaged in a coordinated action may share one or more joint forward models to regulate their actions (Sänger et al., 2011). If interacting dyads predict and monitor the sensory outcomes of both partners’ actions and make adjustments based on both action outcomes, one would expect interpersonal coupling dynamics to emerge during joint actions. Indeed, studies comparing *intra-*personal coordination to *inter*personal coordination showed that both cases rely on the same dynamical organizing principles (Schmidt and Richardson, 2008), in the sense that the limbs of two different persons, just as the different limbs of one person, form a coupled unit. Schmidt and Richardson (2008) suggested that the organizing principles predicted by the coordination dynamics approach “can operate in neurally based behavioral oscillatory systems that are coupled by perceptual information and, consequently, that these principles represent a universal self-organizing strategy that occurs at multiple scales of nature.”

Recent interaction experiments using EEG-hyperscanning, the simultaneous recording of multiple persons’ EEG signals (Babiloni et al., 2007a), suggest that these organizing principles extend to the neural level in the form of inter-brain oscillatory couplings (Sänger et al., 2011; Konvalinka and Roepstorff, 2012). Various forms of interpersonally coordinated actions, such as guitar play (Lindenberger et al., 2009; Sänger et al., 2012), gesturing (Dumas et al., 2010) or romantic kissing (Müller and Lindenberger, 2014), were associated with inter-brain synchronization processes predominantly in frequencies below 20 Hz and between fronto-central and parietal sites. Similarly to studies using EEG-hyperscanning, inter-brain synchronized processes have also been observed using the fMRI- and fNRIS-hyperscanning techniques (for review see Babiloni and Astolfi, 2014). Joint action appears to be consistently characterized by changes in inter-brain coupling dynamics, although few studies have attempted to distinguish neural synchronization processes that reflect shared perceptual input and synchronized motor output from those that reflect the emergence of supra-personal coupling processes (Konvalinka and Roepstorff, 2012). Particularly, it remains unclear if inter-brain dynamics causally contribute to joint action performance or if they merely reflect successful action coordination, given that similarities in perceptual input and motor output of two interacting agents tend to be highest when the agents successfully synchronize their actions (Lindenberger et al., 2009; Dumas et al., 2010). To test the existence of a causal nexus between neural and behavioral between-person coupling phenomena, it is desirable to gain greater experimental control over the degree to which oscillatory activity is synchronized across brains.

### Transcranial Alternating Current Stimulation

Transcranial alternating current stimulation (tACS) seems well suited for this purpose. A growing body of studies has shown that tACS is able to modify cortical excitability and activity as well as behavioral performance in various domains, such as memory, learning, or motor function (Antal and Paulus, 2013; Herrmann et al., 2013). Despite increasing interest in the technique, tACS is still in its beginnings, and its precise working mechanisms are still debated (Reato et al., 2013). Using intracranial recordings in animals, Frohlich and McCormick (2010) demonstrated that an electrical field can entrain neuronal firing. Ozen et al. (2010) added that weak electrical currents can also penetrate skull bones and entrain neuronal firing. Though these data suggest that tACS effects may reflect neural entrainment (Herrmann et al., 2013), the precise operation of tACS remains unclear (Thut et al., 2017) and further the efficiency of tACS remains under strong debate (Horvath et al., 2015; Kleinert et al., 2017). Hence the results of the present study need to be interpreted in the context of these ambiguities.

Overall, there is consensus in the literature that tACS affects local and possibly remote oscillatory activity. Applying tACS at frequencies in the EEG range entrains neuronal networks at the applied frequency (Antal and Paulus, 2013; Herrmann et al., 2013), although Kanai et al. (2008) suggested that frequency dependency of tACS is caused by interactions with ongoing oscillatory activity in the stimulated cortex. The capacity of tACS to increase endogenous brain oscillations at the stimulated frequency has been demonstrated in a study for alpha oscillations (Zaehle et al., 2010). TACS applied at alpha and high gamma frequencies over the somatosensory cortex elicits tactile sensations in a frequency-dependent manner (Feurra et al., 2011). Furthermore, such targeting of specific EEG frequency ranges has been shown to enhance performance in the associated cognitive domains. For example, tACS in the alpha range over visual cortex improved performance in a visual conjunction search (Muller et al., 2015). Notably, Polania et al. (2012) demonstrated that 6 Hz tACS applied in-phase at frontal and parietal sites boosted reaction times in a working memory task, while 180° out-of-phase 6 Hz tACS did not. This study provided proof of concept that tACS can be used to modulate intra-brain synchronized networks and the differences between in- and out-of-phase tACS modulation can impact behavior.

### Hyper-tACS as a Means to Manipulate Inter-Brain Synchronization

In the present study, we adapted the logic of the Polania et al. (2012) study to inter-brain synchronized networks. Instead of modulating the oscillatory phase between stimulation electrodes on one head and thus boosting or disrupting intra-brain synchronized oscillations, we applied tACS simultaneously to two individuals (hyper-tACS) to modulate frequency and phase between stimulation electrodes on two heads. In this manner, we hoped to boost or disrupt inter-brain synchronized oscillations, and examine the effect of this manipulation on the degree of behavioral synchronization. We hypothesized that if inter-brain oscillatory couplings are indeed constitutive for joint action, experimental modulation of inter-brain oscillatory synchronization would affect the degree of interpersonal action coordination.

In order to exert a high degree of experimental control while maintaining the ecological validity and continuous interaction of musical performance paradigms (Acquadro et al., 2016), we used a dyadic drumming paradigm previously established in our lab (Kleinspehn-Ammerlahn et al., 2011). The paradigm was originally derived from the tapping paradigm that has been widely used in the literature to study individual [for review see (Repp, 2005; Repp and Su, 2013)] and more recently also dyadic sensorimotor synchronization abilities (Konvalinka et al., 2010; Vesper and Richardson, 2014). In the classic tapping paradigm, subjects are instructed to tap with their index finger in synchrony with a metronome. Synchronization accuracy is measured as the temporal distance between the finger tap and the metronome click. In dyadic tapping, dyads are instructed to tap symmetrically in synchrony with each other (Konvalinka et al., 2010). One advantage of drumming over tapping is the relatively weaker importance of physical constraints (e.g., finger length) and differences in fine motor skills. Informed by the literature and by the findings in our previous EEG-hyperscanning studies during joint action, we decided to apply tACS at fronto-parietal sites in the theta frequency range over the right hemisphere to target higher-order prediction processes rather than motor processes.

Very recently, Novembre et al. (2017) followed the same logic presented here and applied hyper-tACS during a dyadic finger tapping task. The authors targeted left centroparietal areas at beta frequency to interfere with synchronization processes in motor regions specifically and indeed report facilitation of early inter-personal action synchronization in a same-phase-same-frequency relative to different-phase-same-frequency.

However, inter-brain coupling at *right* centroparietal sites with a topography similar to neuroanatomical sources within the human mirror neuron system (Tognoli et al., 2007) has been observed repeatedly (Tognoli et al., 2007; Dumas et al., 2010) during interpersonal action coordination. This right-lateralized, centroparietal coupling in the alpha-mu range (8–12 Hz), the so-called ‘phi complex,’ has been put forward as a ‘neuromarker for human social coordination’ (Tognoli et al., 2007). Notably, the phi complex has been repeatedly observed during an imitation paradigm that involved moving the left as well as the right hand. Thus, the lateralization of the phi complex to the right hemisphere appears independent of motor behavior and instead might reflect the lateralization of mechanism that support coordinated behavior. Specifically, the phi complex has been proposed to reflect ‘the influence of the other on a person’s ongoing behavior’ (Tognoli et al., 2007, p. 8190). Other authors associated oscillations in right centroparietal areas in a broader frequency range (5–15 Hz)^1^ with self-other integration (Novembre et al., 2016). Within the context of our paradigm it is of interest that rehearsal mechanisms of rhythmic patterns have been suggested to also reside in the right hemisphere (Riecker et al., 2002). Studies in our own lab showed strongest inter-brain synchronization at frontocentral and centroparietal regions predominantly in the delta and theta ranges during joint guitar play (Lindenberger et al., 2009; Sänger et al., 2012; Müller and Lindenberger, 2014). Strongest inter-brain synchronization effects in the theta range for centroparietal and frontocentral connections have also been reported for cooperation in the prisoner’s dilemma (Astolfi et al., 2011). Hence, for the present exploratory study, we chose to integrate these various findings from the literature and opted for a stimulation setup similar to the one previously used by Polania et al. (2012).

### Contribution and Hypotheses of the Study

In this study we applied hyper-tACS during dyadic drumming to manipulate ongoing inter-brain synchronization to study the effect of this manipulation on interpersonal action synchronization. We hypothesized that if inter-brain oscillatory couplings are indeed constitutive for joint action, experimental modulation of inter-brain oscillatory synchronization would affect the degree of interpersonal action coordination.

In particular, we hypothesized that same-phase-same-frequency hyper-tACS would improve dyadic drumming synchronization, while different-phase-different-frequency hyper-tACS would harm dyadic drumming synchronization. Moreover, we also expected that hyper-tACS would not affect behavioral performance when synchronizing to a metronome, as our stimulation did not target motor processes in the left hemisphere, but coordination processes assumed to reside in the right hemisphere. We furthermore included metronome frequencies harmonic to the stimulation frequencies to control for any potential purely motor impact of the tACS on drumming performance at corresponding harmonic frequencies.

## Materials and Methods

### Participants

Initially, 44 female–female dyads participated in the study. Six of the 44 dyads discontinued the experiment, for reasons unrelated to drumming or synchronization performance. Thus, the effective sample consisted of 38 female–female dyads (age range: 20–30 years, mean = 24 years, standard deviation = 2.8 years). Participants did not know each other prior to the study. We decided to include only female participants to prevent effects due to differences in the sex composition of the dyads (Schmid Mast, 2004), as gender distribution in dyads has been found to substantially influence interbrain connectivity patterns (Baker et al., 2016). All participants were right-handed and had normal hearing, full functional mobility in both hands, and no prior musical training. None of the participants suffered from any neurological or psychological disorder, or took medication regularly or during the time the experiment was conducted. Additionally, all of the participants were blind to the hypotheses and conditions of the study.

All participants volunteered for the experiment and gave written informed consent prior to their inclusion in the study. The Ethics Committee of the German Psychology Society approved the study. The study was performed in accordance with the ethical standards laid down in the 1964 Declaration of Helsinki.

### Experimental Setup

Participant pairs were seated back-to-back in an electromagnetically shielded cabin with a portable wall separating both participants. This setup was used to exclude non-verbal communication cues and to allow a relatively tight control of interaction parameters, as the entire flow of information within the dyad was contained in the temporal distribution of the drum beats. Both participants drummed with the drumsticks in their right hands. Drum beats were digitized (Roland drum computer, Germany), and along with auditory instructions and metronome beats (both sent from Intel Xeon, 3.7 GHz PC running Windows 7) played to participants through in-ear headphones, covered by additional soundproof headphones. Drum beat data was recorded from two redundant sources. First, sensors (BIOVISION; single axis, sensitivity: 50 g) attached to the top end of the drumsticks recorded drumstick acceleration, and a peak detection algorithm was used to determine at which exact time points (in milliseconds) drum beats occurred. Second, the digitized drum beat signals were recorded directly via an ExG bipolar amplifier (Brain Products, Munich, Germany) on a second PC (Intel Core i5, 3.2 GHz running Windows XP). Due to technical problems with the acceleration sensors and the higher accuracy of the auditory signal, only the drum beat time series derived from the digitized drum beats were used for further analyses. TACS electrodes were placed first and EEG-electrodes were placed on all sites of a 32-electrodes setup according to the international 10–20 system that were not covered by the tACS electrodes. EEG was thus recorded from both participants with active 21 Ag/AgCl electrodes per person, with the reference electrode at the right mastoid (actiCAP, Brain Products, Munich, Germany). EEG data were collected for a different study, for the present report no EEG data have been analyzed as removal of the tACS-induced EEG-artifact is non-trival.

### Dyadic Drumming Paradigm

**FIGURE 1:**
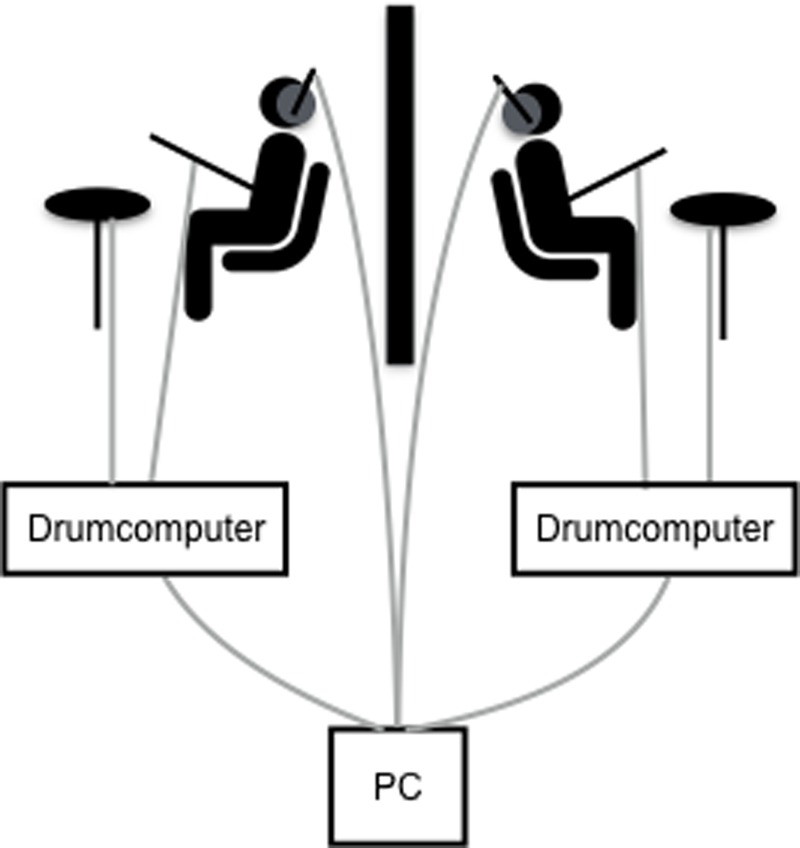
Experimental set-up of the dyadic drumming paradigm. Participants sat back-to-back, were separated by a portable wall and drummed in symmetrical synchrony with each other or with a metronome while receiving hyper-tACS. Drum beats were digitized via the drum computer and along with auditory instructions and metronome beats sent from the PC to the participants’ headphones. Acceleration sensors were placed on the drum sticks. Cable connections depicted in gray.

The dyadic drumming paradigm used in this study (see **Figure 1**) has been previously established in our lab (Kleinspehn-Ammerlahn et al., 2011). The study comprised three different behavioral conditions (dyadic, metronome, and individual drumming), which were delivered in a pseudorandom trial order. Participants were instructed to hold the drumming frequency stable within any given trial. For individual trials each participant was asked to drum at a freely chosen frequency and participants only heard their own drumbeats. *Individual* was chosen to assess each participant’s preferred drumming tempo. For *metronome* trials, participants were asked to drum as precisely as possible in synchrony with a metronome. The metronome beat was varied in a pseudorandom order within and between participants at 1.25, 1.5, or 1.75 Hz. Participants heard both their own drumbeats and the metronome beats. This condition was used to assess each participant’s general synchronization ability. For *dyadic*, participants were asked to drum as precisely as possible in synchrony with each other. Participants heard their own and their partner’s drumbeats. This condition was used to assess mutual synchronization within the dyad and each dyad’s preferred tempo.

All instructions and drum/metronome beats were delivered through headphones. Each trial was prefaced with a word that indicated the condition of the following trial (‘joint’/‘metronome’/‘individual’), followed by a beep that signaled the trial start. After 16 s trial duration, the trial end was signaled by another beep. Subsequently, participants replied to the post-trial questions by button presses. There was one post-trial question for both non-dyadic conditions ‘How stable was the frequency of drumming?’ for individual trials (1 = bad, 2 = ok, 3 = good) and ‘How well did the synchronization go?’ for metronome trials (1 = bad, 2 = ok, 3 = good). After dyadic trials, participants answered two questions: ‘How well did the synchronization go?’ (1 = bad, 2 = ok, 3 = good) and ‘Who determined the frequency of drumming?’ (1 = me, 2 = both 3 = other).

To assess the influence of hyper-tACS on dyadic, metronome and individual drumming we conducted a multiple-session study. Each dyad visited the lab for three experimental sessions, separated by 1 week each. Experimental sessions differed only in hyper-tACS parameter and each session consisted of three segments: pre-stimulation (pre), hyper-tACS stimulation (stimulation), and post-stimulation (post). The pre and post segments were identical and consisted of 21 trials each (five individual, eight dyadic, eight metronome; dyadic and metronome trials alternated in blocks of four trials). To maximize dyadic drumming trials during the stimulation segment, this segment consisted of 45 trials (seven individual, thirty dyadic, and eight metronome; blocks of three dyadic trials were separated by one individual/metronome trial in a pseudorandomized order). Session length varied slightly depending on each dyad’s speed of answering the post-trial questions. Pre and post lasted 10–12 min each and stimulation lasted 21–25 min (variance is due to dyad’s differential response speed to post-trial questions). Participants took a short break between pre and stimulation and between stimulation and post.

### Measures of Behavioral Performance

We used a measure of dyadic drumming asynchrony previously established in our lab, referred to as ‘asynchrony score’ (for details, see Kleinspehn-Ammerlahn et al., 2011, and Supplementary Material). The measure compares synchrony mismatch between two time series of drumbeats. ‘Dyadic asynchrony scores’ were computed by calculating the distance between the series of both participants’ drumbeats as costs of transforming one series into the other to reach perfect synchrony, and ‘metronome asynchrony scores’ by analogously comparing one participant’s drum beat time series with the corresponding metronome beat time series. The transformation was achieved by either shifting drumbeats to later or earlier points in time or by inserting or deleting drumbeats. Using dynamic programming and by pairing drumbeats in a way that an optimal trade-off between shifting and inserting missing drumbeats was assumed, the algorithm automatically minimized the cost function. Transfer costs are expressed in milliseconds and indicate the duration of the needed time shifts and the additional costs for insertion or deletion of drumbeats, which corresponded to half the mean drumbeat interval of the series in question (see Kleinspehn-Ammerlahn et al., 2011 for a formal description of the asynchrony score algorithm). The measure was chosen for its methodological advantages over more traditional metrics, most importantly its independence from speed changes and its ability to match corresponding taps. The measure has a minimum score of zero at perfect synchrony. Asynchrony sum scores were calculated for each trial. To approximate a normal distribution outliers were removed (>2.5 standard deviation) and asynchrony scores were Lambert-transformed using *R* (R Development Core Team, 2008) and the ‘LambertW’ package (Goerg, 2011). Preferred drumming tempo was measured as mean inter-response interval in ms for each trial and also Lambert-transformed to approximate a normal distribution. The Lambert-transformed dyadic asynchrony scores, metronome asynchrony scores and individual and dyad preferred tempo served as the dependent variables in this study.

### Hyper-tACS Protocols

Electrical stimulation was delivered through a four-channel direct current stimulator (DC-Stimulator MC; NeuroConn GmbH, Ilmenau, Germany). The tACS stimulator was connected to three conductive rubber electrodes (each 5 cm × 5 cm). Similar to the setup used by Polania et al., 2012^2^, on each subject’s right hemisphere two stimulation electrodes were placed on F4 (fronto-central) and P4 (parieto-central) of the international 10–20 system. As a multichannel stimulator system was used, each stimulation electrode was connected to one independent channel and both cables of these corresponding return channels were electromechanically soldered into one single merged cable for the return electrode, which was placed on Cz (central). Analog to the protocol used by Polania et al. (2012) stimulation intensity was set to 1mA (peak to peak). The stimulation was automatically ended after 25 min to remain with the range considered safe for use of tACS (see e.g., Antal and Paulus, 2013). In order to apply tACS without irritating the skin under the electrodes, impedance between the electrodes was kept below 20 kOhm throughout the experiment. This was obtained by applying Ten20 conductive gel on the rubber electrodes and onto the hair and skin on the scalp. Also, in order to minimize the sensation caused by sudden stimulus onset, the stimulation intensity was ramped up to the maximum intensity of 1 mA over 30 s and ramped off to zero for 30 s after the stimulation. The sensation on the scalp faded over the initial 1st minute presumably due to adaptation of the skin and the decrease of the impedance. Three different stimulation parameters were used for each dyad in a pseudo-randomized cross-over design (see **Table 1**) that enabled us to control for training effects across sessions. All stimulations were alternating current sinusoidal stimulation within the theta range: (a) ‘same-phase-same-frequency stimulation’: both subjects received stimulation at 6 Hz with a zero phase difference; (b) ‘different-phase-different-frequency stimulation’: one subject received 5 Hz with 13 degrees offset, the other 7 Hz with 1 degree offset; (c) ‘sham stimulation’: both subjects received 30 s fade-in and 30 s fade-out of 6 Hz stimulation (Polania et al., 2012). We selected these frequencies so that both stimulation types remained within the theta range, while at the same time the different-frequencies-stimulation used two prime numbers (5 and 7) as the stimulation frequencies so that one stimulation frequency was not a multiple of the other. Current intensity and the frequencies used in this study were chosen to be unlikely to induce perception of phosphenes usually induced by higher frequencies (Kanai et al., 2008). After each stimulation session, subjects filled out a tACS post-questionnaire (Poreisz et al., 2007), which confirmed the absence of phosphenes in this study. Furthermore, none of the subjects experienced lasting discomfort throughout the experiment.

**Table 1:** Organization of different stimulation types across sessions.

Group	Session 1	Session 2	Session 3
A (13 dyads)	Different	Sham	Same
B (13 dyads)	Same	Different	Sham
C (12 dyads)	Sham	Same	Different

*The different stimulation conditions were pseudo-randomized across the three experimental sessions in a cross-over design [same-phase-same-frequency: tACS with 6 Hz on both subjects; different-phase-different-frequency: tACS with 5 Hz on one and 7 Hz on the other subject; sham: 6 Hz sham tACS]. Stimulation types: different, different-phase-different-frequency stimulation; same, same-phase-same-frequency stimulation; sham, sham stimulation.*

### Statistical Procedures

We used (R Development Core Team, 2008) and *lme4* (Bates et al., 2015) to perform a linear mixed effects analysis of the relationship between behavioral drumming performance and stimulation type. We constructed four separate models with (a) dyadic asynchrony, (b) metronome asynchrony, (c) individual preferred tempo, and (d) dyad preferred tempo as dependent variables. As fixed effects, we entered condition (1:7), which was a combination of stimulation type (sham; same-phase-same-frequency; different-phase-different-frequency) and experimental segment (pre-stimulation, stimulation, post-stimulation): (1) pre-stimulation, (2) sham stimulation, (3) same-phase-same-frequency stimulation, (4) different-phase-different-frequency stimulation, (5) sham post-stimulation, (6) same-phase-same-frequency post-stimulation, and (7) different-phase-different-frequency post-stimulation. Dyad-level intercepts and by-dyad slopes of drumming exposure in weeks were considered random effects [behavioral performance ∼ condition (1+ drumming exposure in weeks| dyad)]. We used *MASS* (Venables and Ripley, 2002) to create customized contrast matrices to directly compare conditions of interest.

Visual inspection of residual plots did not reveal any obvious deviations from homoscedasticity or normality. *P*-values for the effect of condition were obtained by likelihood ratio tests of the full model with the effect in question [behavioral performance ∼ condition (1+ drumming exposure in weeks| dyad)] against the model without the effect in question [behavioral performance ∼ (1+ experimental session| dyad)]. *P*-values for all individual factor levels of the fixed effects were calculated from *F* statistics of types I–III hypotheses using Satterthwaite’s approximation for denominator degrees of freedom. The tests on random effects were performed using likelihood ratio tests (both implemented in *R* statistical software using ‘lmerTest’). We further used a series of Welch’s unequal variance *t*-tests to analyze mean differences between individual and preferred tempo and an *F*-test to analyze differences in variance. To specifically test the relationship between individual preferred tempo and dyad preferred tempo we performed another linear mixed effects analysis. The dependent variable was dyad preferred tempo, as fixed effects we entered individual A preferred tempo and individual B preferred tempo, random effects were again intercepts for dyads and by-dyad random slopes for the effect of week.

## Results

### Dyadic Asynchrony

**FIGURE 2:**
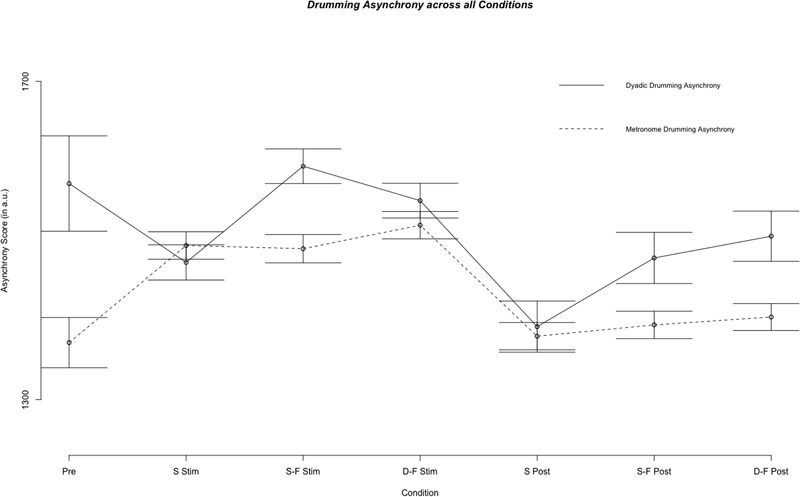
Results of the fixed effect of the linear mixed model analysis for the effect of condition on dyadic and metronome asynchrony scores. Displayed are mean asynchrony scores as estimated by the model. Stimulation conditions: Pre, pre; S Stim, sham stimulation; S-F Stim, same-phase-same-frequency stimulation; D-F Stim, different-phase-different-frequency stimulation; S Post, sham post; S-F Post, same-phase-same-frequency post; D-F Post, different-phase-different-frequency post. Standard errors are indicated by horizontal lines.

**FIGURE 3:**
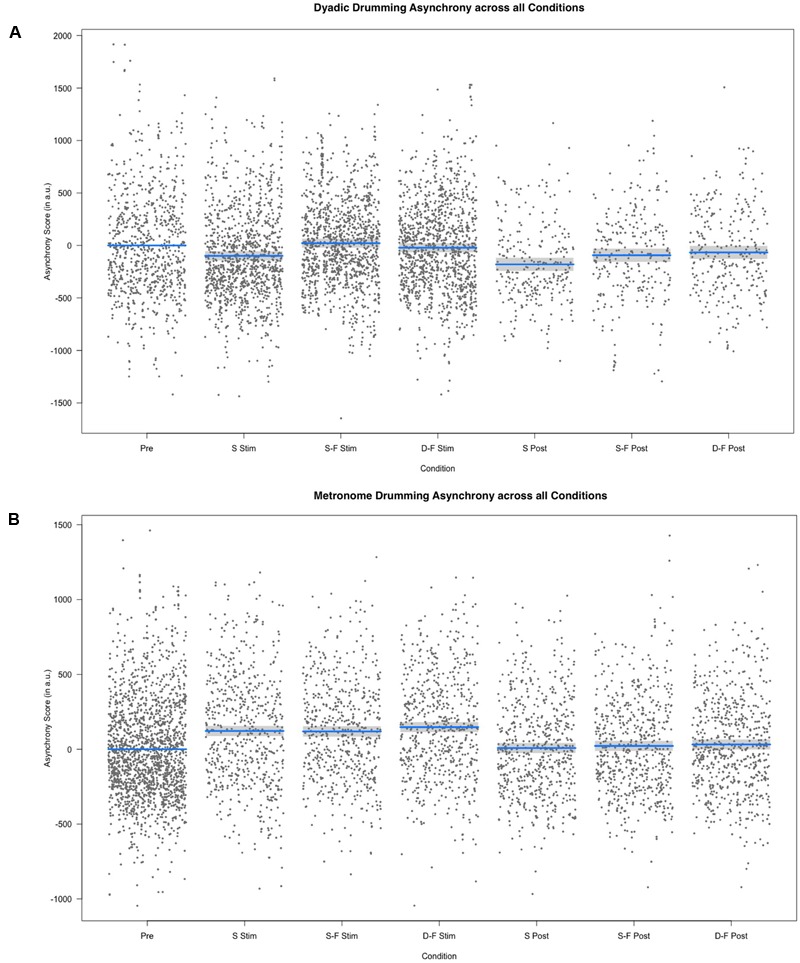
Results of the fixed effect of the linear mixed model analysis for the effect of condition on asynchrony scores. **(A)** Partial residuals as estimated by the mixed model for dyadic drumming. **(B)** Partial residuals as estimated by the mixed model for metronome drumming. Stimulation conditions: Pre, pre; S Stim, sham stimulation; S-F Stim, same-phase-same-frequency stimulation; D-F Stim, different-phase-different-frequency stimulation; S Post, sham post; S-F Post, same-phase-same-frequency post; D-F Post, different-phase-different-frequency post. Blue lines indicate model prediction values. 95% confidence intervals are displayed as gray bands.

Linear mixed model analysis showed differential effects for conditions (combination of stimulation type and experimental segment) on dyadic asynchrony [χ^2^(6) = 60.21, *p* < 0.001]. Relative to pre-stimulation, same-phase-same-frequency stimulation and different-phase-different-frequency stimulation did not change dyadic asynchrony [*t*(4856) = 0.872, *p* = 0.383; *t*(4868) = -1.444, *p* = 0.149], while dyadic asynchrony decreased for sham stimulation and all post-stimulation conditions (all *p* < 0.05, see **Figures 2, 3**). We estimated the regression model with customized contrast matrices to compare sham stimulation to the mean of same-phase-same-frequency stimulation and different-phase-different-frequency stimulation. Relative to sham stimulation, dyadic asynchrony increased under active stimulation [β = 84.72, *SE* = 21.89, *t*(4499) = 3.870, *p* < 0.0005]. Relative to same-phase-same-frequency stimulation, different-phase-different-frequency stimulation decreased dyadic asynchrony [β = -49.50, *SE* = 24.14, *t*(4791) = –2.050, *p* < 0.05]. Relative to sham post-stimulation the mean dyadic asynchrony across different-phase-different-frequency post-stimulation and same-phase-same-frequency post-stimulation was increased [β = 78.63, *SE* = 34.34, *t*(4890) = 2.290, *p* < 0.05]. We observed no difference when comparing different-phase-different-frequency post-stimulation directly to same-phase-same-frequency post-stimulation [*t*(5904) = 0.419, *p* = 0.675]. Random effects accounted for 72.30% of variance in dyadic asynchrony scores.

### Metronome Asynchrony

Linear mixed model analysis showed differential effects for conditions (combination of stimulation type and experimental segment) on metronome asynchrony [χ^2^(6) = 146.65, *p* < 0.001]. Relative to pre-stimulation, metronome asynchrony was increased in all three stimulation conditions, but in no post-stimulation condition (all *p* > 0.05, see **Figures 2, 3**). In analogy to the analysis of dyad asynchrony we used customized contrast matrices to compare sham and active tACS conditions directly. Metronome asynchrony during same-phase-same-frequency stimulation did not differ from metronome asynchrony during different-phase-different-frequency stimulation [*t*(4830) = 1.434, *p* = 0.152]. Further, metronome asynchrony during sham stimulation was not different from mean metronome asynchrony across same-phase-same-frequency stimulation and different-phase-different-frequency stimulation [*t*(4607) = 0.474, *p* = 0.636]. Same-phase-same-phase-same-frequency post-stimulation was not different from different-phase-different-frequency post-stimulation [*t*(4730) = 0.436, *p* = 0.663], neither was metronome asynchrony during sham post-stimulation different from mean metronome asynchrony across same-phase-same-frequency post-stimulation and different-phase-different-frequency post-stimulation [*t*(4570) = 0.835, *p* = 0.404]. The random effects accounted for 48.92% of total variance in metronome asynchrony. Whether or not the metronome frequency was harmonic to the stimulation frequency did not effect metronome asynchrony score. A mixed model with metronome frequency, stimulation frequency and harmonic (as a factor) did not perform better than a model containing only the random effects subject id and weeks of drumming exposure [χ^2^(3) = 0.205, *p* = 0.977].

### Direct Comparison of Dyadic and Metronome Asynchronies

**FIGURE 4:**
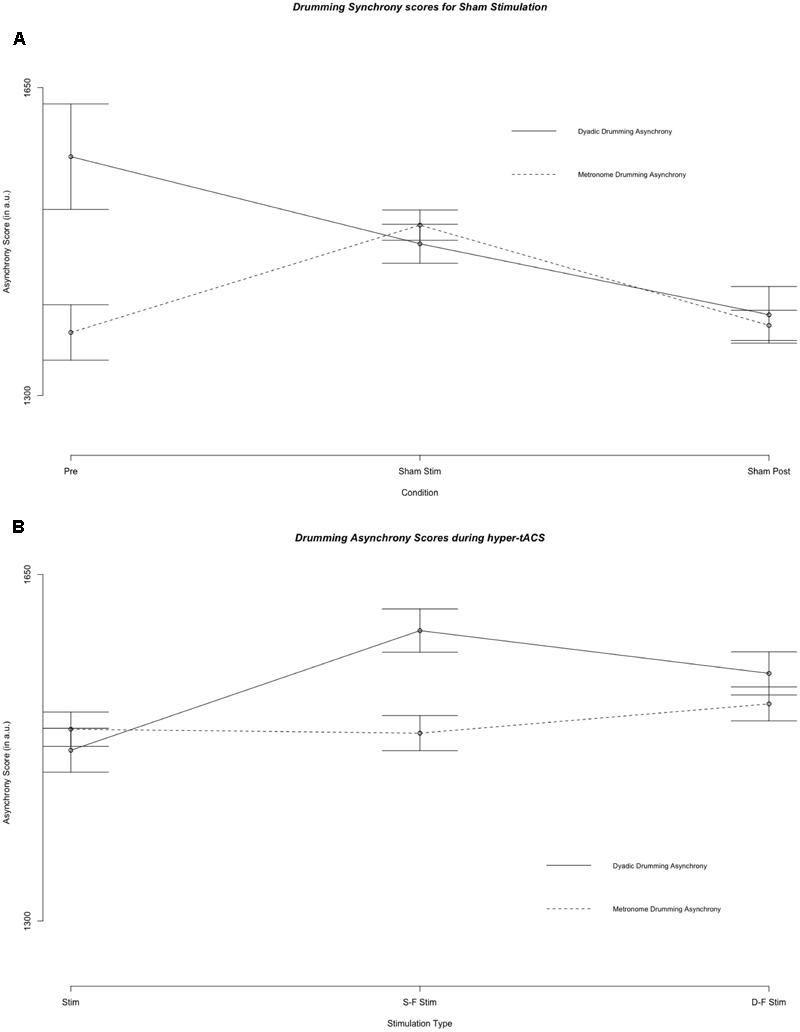
Results of the fixed effect of the linear mixed model analysis for the effect of condition on asynchrony scores. **(A)** Asynchrony scores during pre stimulation (Pre), sham stimulation (S Stim) and sham post-stimulation (S Post). **(B)** Asynchrony scores during the three stimulation types: sham stimulation (S Stim), same-phase-same-frequency stimulation (S-F Stim) and different-phase-different-frequency stimulation (D-F Stim). Standard errors are indicated by horizontal lines.

**Figure 2** illustrates mean metronome asynchrony scores for all experimental conditions. A direct comparison between dyadic asynchrony and metronome asynchrony for sham tACS revealed a striking similarity between metronome asynchrony scores and dyadic asynchrony scores (see **Figure 4**). For sham tACS, participants were better at synchronizing with a metronome only at the beginning of the experiment (pre-stimulation) [Welch’s unequal variance *t*-test: *t*(1253) = 10.528, *p* < 0.001], while they performed just as well in dyadic drumming during the experimental segments stimulation [*t*(1536) = -0.139, *p* = 0.89] and post-stimulation [*t*(467) = 0.569, *p* = 0.570]. Overall, dyads improved in dyadic synchronization over the course of the experimental session (pre-stimulation > stimulation > post-stimulation), while participants’ synchronization to the metronome was best during pre-stimulation (see **Figures 2, 4**). The linear mixed model analyses showed a differential impact of tACS on metronome asynchrony scores vs. dyadic asynchrony scores. **Figure 4** visualizes the impact of sham tACS and the two active stimulation protocols onto dyadic asynchrony vs. metronome asynchrony: mean metronome asynchrony scores [averaged across all three experimental segments (pre-stimulation, stimulation, post-stimulation)] and mean dyadic asynchrony scores were similar for sham tACS. Only mean metronome asynchrony scores remained stable for the two active stimulation protocols, while mean dyadic asynchrony scores were increased for both active stimulation protocols.

### Individual Preferred Tempo

**FIGURE 5:**
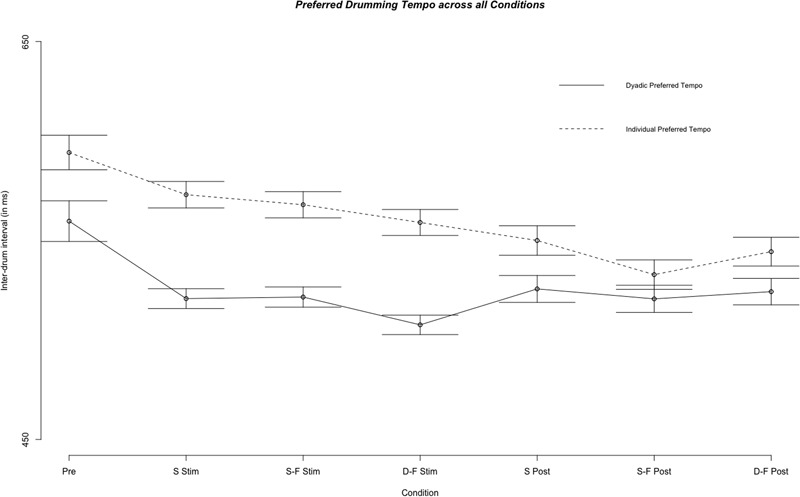
Comparison of the fixed effect results of the linear mixed model analysis for the effect of condition on individual preferred and dyadic preferred drumming tempo for all experimental conditions. Displayed are mean inter-drum intervals as estimated by the model. Stimulation conditions: Pre, pre; S Stim, sham stimulation; S-F Stim, same-phase-same-frequency stimulation; D-F Stim, different-phase-different-frequency stimulation; S Post, sham post; S-F Post, same-phase-same-frequency post; D-F Post, different-phase-different-frequency post. Standard errors are indicated by horizontal lines.

**FIGURE 6:**
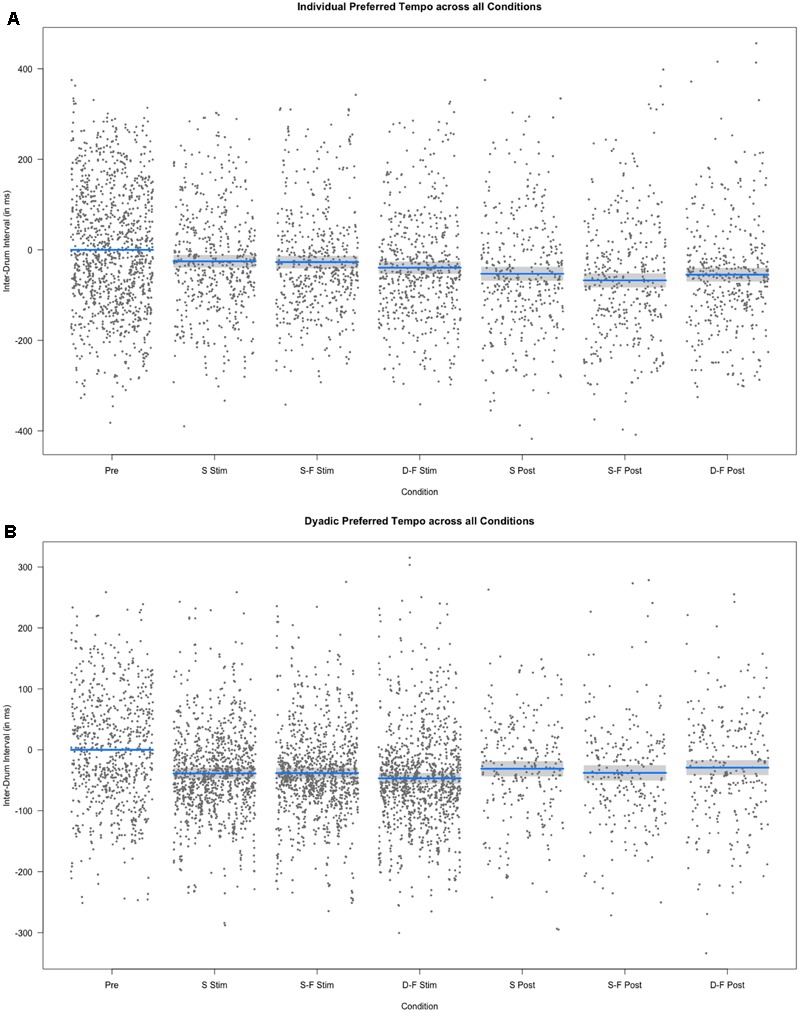
Results of the fixed effect of the linear mixed model analysis for the effect of condition on individual and dyadic preferred drumming tempo. **(A)** Partial residuals as estimated by the mixed model for individual preferred drumming tempo. **(B)** Partial residuals as estimated by the mixed model for dyadic preferred drumming tempo. Stimulation conditions: Pre, pre; S Stim, sham stimulation; S-F Stim, same-phase-same-frequency stimulation; D-F Stim, different-phase-different-frequency stimulation; S Post, sham post; S-F Post, same-phase-same-frequency post; D-F Post, different-phase-different-frequency post. Blue lines indicate model prediction values. 95% confidence intervals are displayed as gray bands.

Mean individual preferred inter-tap interval across all experimental segments and sessions was 593.21 ms (*SE* = 237.31 ms) and did not differ from the mean across sham trials [*M* = 591.05 ms, *SE* = 230.83 ms, Welch’s unequal variance *t*-test: *t*(678) = 0.200, *p* = 0.841]. However, individual preferred tempo for pre-stimulation trials was different from the tempo across all preferred tempo trials [*M* = 637.64 ms, *SE* = 255.55 ms, Welch’s unequal variance *t*-test: *t*(1779) = -5.261, *p* < 0.0001]. Linear mixed model analysis showed differential effects for conditions (combination of stimulation type and experimental segment) on individual preferred tempo [χ^2^(6) = 118.54, *p* < 0.0001, see **Figures 5, 6**]. Relative to pre-stimulation, individual preferred inter-tap interval was decreased in all other conditions (all *t* > 3.488, *p* < 0.0001). Customized contrasts showed no difference in individual preferred tempo between same-phase-same-frequency stimulation and different-phase-different-frequency stimulation [*t*(2854) = -01.618, *p* = 0.105], nor a difference in individual preferred tempo between sham stimulation and the mean across both active tACS stimulations [*t*(2571) = -0.656, *p* = 0.512].

Preferred tempo also did not differ between same-phase-same-frequency and different-phase-different-frequency post-stimulation [*t*(3295) = -0.399, *p* = 0.690], neither differed it between sham post-stimulation and the mean across same-phase-same-frequency and different-phase-different-frequency post-stimulation [*t*(3104) = -1.371, *p* = 0.170]. The random effects explained 44.87% variance in preferred tempo.

### Dyadic Preferred Tempo

Mean dyad preferred inter-tap interval across all experimental segments and sessions was 515.06 ms (*s* = 138.99 ms) and it was not different from mean across sham trials alone [*M* = 511.57 ms, *s* = 121.34 ms; Welch’s unequal variance *t*-test: *t*(1695) = 0.826, *p* = 0.409]. We tested the difference between overall individual preferred tempo and overall dyadic preferred tempo with a Welch’s unequal variance *t*-test [*t*(5887) = -18.192, *p* < 0.0001]. This difference was also significant when comparing individual and preferred tempo only on trials before any stimulation was applied (pre-stimulation) [*t*(1913) = -9.164, *p* < 0.0001] or when comparing only sham trials [*t*(667) = -7.378, *p* < 0.0001]. Furthermore, variance (Var) was higher for individual preferred tempo than for dyad preferred tempo [Var (individual) = 56317.64, Var (dyad) = 19318.06; *F*(4955) = 0.343, *p* < 0.0001]. See **Figure 5** for a comparison of individual and dyad preferred tempo by condition. Linear regression analysis with the sum of both players’ mean individual preferred tempo and the fastest mean individual preferred tempo as factors showed a predictive effect of the sum on variance in mean dyad preferred tempo [*F*(40) = 10.77, *p* < 0.0005, *R*^2^ adjusted = 0.317; sum: *t*(40) = 2.056, *p* < 0.05)]. Linear mixed model analysis showed differential effects for conditions (combination of stimulation type and experimental segment) on dyad preferred tempo [*χ*^2^(6) = 163.13, *p* < 0.0001]. Relative to pre-stimulation, dyad preferred inter-tap interval was decreased in all other conditions (see **Figures 5, 6**). Neither same-phase-same-frequency stimulation nor different-phase-different-frequency stimulation differed from sham stimulation [*t*(2910) = 0.931, *p* = 0.352; *t*(3164) = -0.667, *p* = 0.505]. The same was true for same-phase-same-frequency post-stimulation, different-phase-different-frequency post-stimulation and sham post-stimulation [*t*(4610) = -0.415, *p* = 0.678; *t*(4714) = 0.107].

## Discussion

### General Discussion

The aim of the present study was to investigate how manipulation of ongoing inter-brain phase synchronization by hyper-tACS would affect the synchrony of dyadic drumming performance. Previous research showed that the real-time neural dynamics of various forms of interpersonally coordinated behavior are characterized by inter-brain phase synchronization (Konvalinka and Roepstorff, 2012). To our knowledge, and with the notable exception of Novembre et al. (2017, see below), previous studies that investigated inter-brain dynamics during interpersonal action coordination were observational in nature. The present study is an attempt to manipulate inter-brain dynamics and examine the effects of this manipulation on interpersonal action coordination.

The main result of the present study is that, compared to sham stimulation, only dyadic asynchrony was modulated by same-phase-same-frequency and different-phase-different-frequency hyper-tACS. Metronome asynchrony, individual preferred tempo and dyad preferred tempo were not modulated. Performance on all four behavioral measures changed between pre-stimulation and stimulation, and between stimulation and post-stimulation conditions under sham hyper-tACS: Dyadic asynchrony decreased while metronome asynchrony increased, and both individuals and dyads preferred faster tempi (see **Figures 2, 5**). We suspect that the decrease in asynchrony for dyadic drumming from pre-stimulation to sham stimulation reflects learning or ‘tuning-in’ processes within a dyad. As subjects were very good at synchronizing to a metronome already in the pre-stimulation condition, the slight decrease in performance over the course of the experiment might reflect a ceiling effect. Under same-phase-same-frequency and different-phase-different-frequency tACS, metronome asynchrony as well as individual and dyad preferred tempo developed just as under sham stimulation, while dyadic asynchrony remained unchanged compared to pre-stimulation and increased compared to sham stimulation. This finding suggests that artificial modulation of naturally occurring inter-brain synchronization in the theta frequency range at frontocentral and centroparietal sites in the left hemisphere during joint action by hyper-tACS may actually impair, rather than improve, dyadic learning. Task difficulty as an alternative explanation for the differential effect of active tACS on dyadic asynchrony and metronome asynchrony is unlikely. Although participants notably synchronized better with a metronome than with each other during pre, this performance difference was no longer present during sham and post-sham. Here, dyadic and metronome synchronization performance were identical (see **Figure 4**). Thus, dyadic synchronization does not appear to be more difficult than metronome synchronization *per se*. Once subjects completed an initial practice period and presumably ‘tuned-in’ to each other, we observed no performance difference between metronome synchronization and dyadic synchronization.

Inter-brain synchronization appears to play a functional role in establishing interpersonally coordinated actions.

Contrary to our guiding hypothesis, we did not observe a differential effect of same-phase-same-frequency and different-phase-different-frequency hyper-tACS on dyadic drumming performance. It is possible that this results from person-to-person variation in the actual frequency of neural entrainment caused by tACS stimulation. It is known that tACS stimulation of a single location may produce a range of different effects at the neural level. For example, 10 Hz tACS applied over the motor cortex inhibits motor evoked potential but improves visuo-motor implicit learning (Antal and Paulus, 2013). More important, the efficacy of tACS depends on the power of endogenous oscillations in the individual’s brain at the targeted frequency (Ruhnau et al., 2016) and the electrode placement protocol used in the present study left room for individual differences in electrical current flow (Datta et al., 2012; Cabral-Calderin et al., 2016). As a result of unique differences within each dyad, we might not have succeeded in boosting inter-brain synchronization precisely. Instead, individual differences in the neuronal response to our same-phase-same-frequency stimulation protocol might have resulted in neuronal entrainment at slightly different frequencies for the two members of the dyad. As a result, our same-phase-same-frequency protocol may in fact have resulted in an out-of-phase, out-of-frequency neuronal response.

We did not observe any performance increases when participants drummed in synchrony with a metronome whose frequency was harmonic to the tACS frequency. This null result might either be taken to support our operationalization to target coordination rather than pure motor processes, or to support the interpretation that hyper-tACS was not successful in boosting the same frequencies in both brains. While “there is need of online tACS/EEG evidence to open a new frontier in oscillatory brain rhythms investigations” (Feurra et al., 2012, p. 2) the separation of tACS artifacts and brain activity in EEG (Helfrich et al., 2014) and MEG signals (Neuling et al., 2015) has only been pioneered recently and there is yet “no established method for precise source localization and artifact-free source reconstruction of tACS-entrained brain oscillations near and underneath the stimulator electrodes” (Witkowski et al., 2016, p. 89; see also Bergmann et al., 2016). Consequently, the present study does not directly assess the efficacy and precision of hyper-tACS in entraining inter-brain oscillations (see Limitations).

Further, correlations between behavioral performance and synchrony in inter-brain dynamics have only been reported in a few studies and often did not follow a linear ‘more is better’ principle. In a study using a turn-based card game paradigm, Babiloni et al. (2007b) reported that only participants belonging to the same team showed functional oscillatory connectivity. The authors also reported directed coherence between activity at frontal sides in the leader’s brain with activity at frontal and parietal sides in the follower’s brain (Astolfi et al., 2010). Such asymmetries in inter-brain dynamics within a dyad were observed in other paradigms too: Konvalinka et al. (2014) collected EEG hyperscanning data during a synchronized finger-tapping task. In contrast to tapping with a metronome, tapping with the other participant coincided with suppressed alpha and low-beta activity over central and frontal areas. In eight out of nine dyads, this suppression of alpha oscillations was more pronounced for the leader than for the follower during both task anticipation and execution. Jiang et al. (2015) assessed the relation between leadership and multibrain dynamics via functional near infrared spectroscopy (fNIRS) hyperscanning in a leaderless group discussion paradigm. The authors reported higher levels of inter-brain synchronization for leader-initiated communications compared to the ones initiated by followers. Sänger et al. (2012) also observed higher within-brain synchrony for the leader as compared to the follower while playing guitar in duet. Taken together, interpersonally coordinated joint action appears to be consistently characterized by changes in inter-brain coupling dynamics; however, in the case of lead-follow behavior these dynamics tend to be asymmetric and non-linear, comparable to dynamics of behavioral synchronization that show significant non-stationarity (Boker et al., 2011). Findings from two recent studies in our lab corroborate the importance of non-linear influences. These studies used graph theory measures to analyze the hyperbrain networks involved in joint guitar play (Sänger et al., 2013) and in romantic kissing (Müller and Lindenberger, 2014). This analysis technique makes it possible to capture more complex aspects of inter-brain dynamics. Sänger et al. (2013) detected different patterns of directed between-brain couplings for leader vs. followers, while Müller and Lindenberger included cross-frequency inter-brain dynamics into the analysis and could show positive as well as negative correlations between measures of inter-brain synchronization strength and kissing satisfaction. Taken together, the stimulation frequencies and topographies needed to facilitate sustained joint action coordination might be more complex and specific than broad right frontoparietal 6 Hz coupling. We included metronome asynchrony and individual dyad preferred tempo as control conditions for the study. As we did not observe any changes related to active tACS in any of the three measures, we conclude that the modulations observed in dyadic asynchrony are not due to a direct interference of tACS with individual motor processes but indeed result from interference of tACS with ongoing inter-brain dynamics. Novembre et al. (2017) recently showed that hyper-tACS applied over left centroparietal areas at 20 Hz improved the synchronization of the first four taps in a dyadic finger tapping task but not in later taps. Thus synchronization processes closer to the motor level appear to indeed have a prominent affect on inter-personal action coordination (initiation), although Novembre et al. (2017) used different stimulation frequencies within one session and thus possible confounds by tACS after-effects (Veniero et al., 2015) from stimulation blocks at 2 and 10 Hz cannot be excluded.

### Individual and Dyad Preferred Drumming Tempi

The range of individual preferred tempo found in the present study is comparable to the range reported in the literature (Fraisse, 1982; Kay et al., 1987; Moelants, 2002). Furthermore preferred tempi increased as a function of time which replicates findings by Collyer et al. (1994), who reported a tendency for individual preferred tempo to increase after a few trials. Distribution of dyad preferred tempo was comparable to the distribution observed with the same paradigm by Kleinspehn (2008).

To our knowledge, dyad preferred tempo, its relation to the tempi preferred by the two individuals within the dyad and its stability over time has not been systematically studied in a tapping or drumming paradigm yet. Interestingly, dyad preferred tempo was generally faster than preferred tempo in individual drumming. Like individual-preferred tempo, it increased after the pre-stimulation, potentially as a function of time (compare 9). Though faster, dyad-preferred tempi were characterized by lower inter-trial variance than individual-preferred tempi. The fact that preferred tempo increased when drumming dyadically may relate to the suggestion that interacting individuals decrease their temporal variability in an effort to make themselves more predictable and thus facilitate joint action (Vesper et al., 2011). The finding that the two individual-preferred tempi within a dyad explained variance in dyad preferred tempo, further corroborates this interpretation that an increase from individual to preferred tempo is not accidental, but mechanistic to dyadic drumming. Interestingly the sum of both individual preferred tempi explained more variance than the difference between individual preferred tempi or the faster/slower tempo alone. This might indicate that it is the interaction between the individuals and not the more dominant individual that gives rise to the speeding phenomenon in dyad preferred drumming tempo. Further research is needed that specifically investigates the mechanistic interplay between individual and dyad preferred tempi.

### Limitations

Due to methodological challenges in controlling current flow and precise neural entrainment with tACS, this present pioneering study lacks a validation to what degree the neural frequencies of the interacting participants become more synchronized or desynchronized upon hyper-tACS. Thanks to recent advances in the field of non-invasive brain stimulation stimulation protocols that circumvent stimulation artifacts have been introduced, such as amplitude-modulated tACS (Witkowski et al., 2016), which allow for source reconstruction and mapping of entrained brain oscillations. Future studies combining hyper-tACS and EEG with refined stimulation protocols (Alam et al., 2016) are needed to overcome this limitation and to extend the findings of this present study. Consequently, the present study is limited in that it does not directly assess the efficacy and precision of hyper-tACS in entraining inter-brain oscillations. Future work using these techniques might be able to more precisely determine the actual induced frequency responses, and be able to account for differential individual responses to the tACS stimulation.

The stimulation protocol chosen further limits this study in that stimulation was delivered only to frontocentral and centroparietal areas in the right hemisphere at specific frequencies in the theta range. Future research is needed to verify if similar results could be obtained with different stimulation frequencies within the theta range. This setup might have prevented us from detecting effects of hyper-tACS on synchronization phenomena closer to the motor level. In a recent study Novembre et al. (2017) reported that hyper-tACS facilitated synchronized interpersonal action initiation, but not sustained action coordination, specifically when applied over centroparietal regions over the left hemisphere at 20 Hz with 0 degree relative phase different, but not when applied at 10 Hz, 2 Hz or with 180 degrees relative phase difference. Future hyper-tACS studies using more complex stimulation protocols targeting for example right centroparietal areas in the theta or mu frequency range (as a ‘marker of social coordination’) and centroparietal areas contralateral to the drumming hand in the beta frequency range (representing networks closer to the motor level) may extend our understanding of how inter-brain synchronization processes facilitate the initiation and sustention of inter-personal action coordination.

## Conclusion

The present study is an attempt to experimentally manipulate inter-brain dynamics and observe the effects of this manipulation on joint action performance. We operationalized this goal by applying same-phase-same-frequency and different-phase-different-frequency hyper-tACS during a dyadic drumming paradigm, where dyads were instructed to drum in synchrony with another participant. Contrary to expectations, we found a reduction in dyadic synchrony during active hyper-tACS when compared to sham tACS. This reduction was not evident when individuals were asked to synchronize to a metronome, nor paralleled by corresponding changes in dyadic drumming frequency or individual preferred tempo.

We suspect that the observed impairment in dyadic drumming synchrony in the same-phase-same-frequency tACS condition may reflect individual differences in the frequency entrainment induced by tACS. Further hyper-tACS studies with more precise stimulation protocols are needed that ensure oscillations in the same frequencies are entrained in the brains of two individuals engaging in joint action.

As a byproduct of the paradigm used, we observed specific relationships between individual and dyad preferred drumming tempi. The tempi preferred by the two individuals in a dyad predicted the dyad’s preferred tempo. However, the dyad’s preferred tempo was generally characterized by lower variance and higher frequencies than the tempi preferred by the individuals alone. The interplay between individual- and dyad-preferred drumming or tapping tempo might present a useful clues for a more mechanistic understanding of interpersonal action coordination.

## Ethics Statement

This study was carried out in accordance with the recommendations of Deutsche Gesellschaft für Psychologie with written informed consent from all subjects. All subjects gave written informed consent in accordance with the Declaration of Helsinki. The protocol was approved by the Deutsche Gesellschaft für Psychologie.

## Author Contributions

CS, TB, VM, and UL designed the experiment. CS acquired the data. CS and TvO analyzed the data. CS, TB, VM, and UL interpreted the data. CS, TB, VM, and UL wrote the manuscript. CS, TB, VM, TvO, and UL approved the final version of the manuscript.

## Conflict of Interest Statement

The authors declare that the research was conducted in the absence of any commercial or financial relationships that could be construed as a potential conflict of interest.

## Footnotes

1 Novembre et al. (2016) defined the alpha range for each participant individually as individual alpha peak power (range: 7–13 Hz) ± 2 Hz.

2 The setup by Polania et al. (2012) has been criticized (Thut et al., 2017) for potentially inducing differences in current flow between the synchronized and desynchronized conditions. In our setup the participant’s two ‘active’ stimulation electrodes were always in-phase and out-of-phase only with respect to the other participant’s stimulation electrodes; thus this limitation did not apply to our setup.
