# Supplementary material for: Hyper-Transcranial Alternating Current Stimulation: Experimental Manipulation of Inter-Brain Synchrony
Source: Front Hum Neurosci. 2017 Nov 8;11:539. doi: 10.3389/fnhum.2017.00539 (PMC5682643; doi:10.3389/fnhum.2017.00539)
Supplement: Supplementary file 1 [file Presentation_1.pdf]

Supplementary Material

Formal Description of Asynchrony Score Algorithm

The asynchrony measure between two drumming series A and B applied in this article is based on the minimal cost to transfer A into B or vice versa. The cost is the sum of all milliseconds that drum beats from series A need to be shifted to match beats from series B. If the distances are too large, beats may be removed or inserted for a fixed cost penalty defined as half the mean drumbeat interval of the series in question. The actual asynchrony value is the cost for the most efficient transformation. In the following, a Java source code is given that computes this transformation. For efficiency, dynamical programming is applied to this problem. A table T of length a of A times the length b of B is initialized; after the algorithm has stopped, the entry (i, j) in the table will give the minimal cost to transform the suffix of A beginning at I to the suffix of B beginning at j. The entry (a1, b1) is initialized at zero. Then, a loop counts down i from l11 to 0 and j from l21 to 0. In each loop, the table entry at (i, j) is computed dependent on the entries in (i1, j1), (i, j1) and (i1, j), which have been computed before. The new entry at (i, j) is the minimum of three possibilities: the entry (i1, j1) plus the distance of the ith beat in A to the jth beat in B, the entry of (i1, j) plus the deletion penalty, or the entry of the (i, j1) plus the deletion penalty. After the loop has ended, the entry at (0,0) is the minimal cost to transfer A to B. The following Java program computes the entries of T and returns the total cost as well as the optimal series of shifts and insertions/deletions:

```
public double[] asynchronity(int[] series1, int[] series2, int penalty) {

  int s1len = series1.length, s2len = series2.length;
  int[][] bestKnownDistance = new int[s1len+1][s2len+1];

   // Computation of Table T
  bestKnownDistance[s1len][s2len] = 0;
  for (int i = s2len–1; i>=0; i--) bestKnownDistance[s1len][i] =
bestKnownDistance[s1len][i+1] + penalty;
  for (int i = s1len–1; i>=0; i--) bestKnownDistance[i][s2len] =
bestKnownDistance[i+1][s2len] + penalty;
  for (int i=s1len–1; i>=0; i--)
    for (int j=s2len–1; j>=0; j--)  bestKnownDistance[i][j] = Math.min(
Math.min(bestKnownDistance[i+1][j],bestKnownDistance[i][j+1]) + penalty,
       bestKnownDistance[i+1][j+1] + Math.abs(series1[i]–series2[j]) );

  int gesValue = bestKnownDistance[0][0];
}
```
